# Agreement between Optoelectronic System and Wearable Sensors for the Evaluation of Gait Spatiotemporal Parameters in Progressive Supranuclear Palsy

**DOI:** 10.3390/s23249859

**Published:** 2023-12-16

**Authors:** Carlo Ricciardi, Noemi Pisani, Leandro Donisi, Filomena Abate, Marianna Amboni, Paolo Barone, Marina Picillo, Mario Cesarelli, Francesco Amato

**Affiliations:** 1 Department of Electrical Engineering and Information Technology, University of Naples Federico II, 80125 Naples, Italy; 2 Department of Advanced Biomedical Sciences, University of Naples Federico II, 80131 Naples, Italy; 3 Department of Advanced Medical and Surgical Sciences, University of Campania Luigi Vanvitelli, 80138 Naples, Italy; 4 Center for Neurodegenerative Diseases (CEMAND), Department of Medicine, Surgery and Dentistry “Scuola Medica Salernitana”, University of Salerno, 84131 Salerno, Italy; 5 Department of Engineering, University of Sannio, 82100 Benevento, Italy

**Keywords:** gait analysis, optoelectronic system, wearable device, spatiotemporal parameters, progressive supranuclear palsy

## Abstract

The use of wearable sensors for calculating gait parameters has become increasingly popular as an alternative to optoelectronic systems, currently recognized as the gold standard. The objective of the study was to evaluate the agreement between the wearable Opal system and the optoelectronic BTS SMART DX system for assessing spatiotemporal gait parameters. Fifteen subjects with progressive supranuclear palsy walked at their self-selected speed on a straight path, and six spatiotemporal parameters were compared between the two measurement systems. The agreement was carried out through paired data test, Passing Bablok regression, and Bland-Altman Analysis. The results showed a perfect agreement for speed, a very close agreement for cadence and cycle duration, while, in the other cases, Opal system either under- or over-estimated the measurement of the BTS system. Some suggestions about these misalignments are proposed in the paper, considering that Opal system is widely used in the clinical context.

## 1. Introduction

Progressive supranuclear palsy (PSP) is a rare neurodegenerative disease characterized by oculomotor gaze palsy, akinesia, postural instability, and cognitive dysfunction. Gait abnormalities represent one of the cornerstones of PSP and determine an increased risk of falls from the earliest stages of the disease [1,2,3]. The PSP Rating Scale (PSP-RS) is a semi-quantitative clinician-based scale considered the gold standard to evaluate disease severity in both clinical and research settings. However, only a few items of the PSP-RS are dedicated to gait and to axial impairment in general [4].

Beyond clinical scales, gait analysis is considered one of the main tools to measure quantitative gait-related parameters [5,6]. In detail, the instrumented gait analysis can assess an individual’s gait patterns through the calculation of quantitative gait-related parameters, namely kinetic, kinematic, and spatiotemporal variables. In neurodegenerative diseases, gait analysis has been used for monitoring disease progression over time, quantifying parkinsonian symptoms [7], evaluating treatments’ outcomes, implementing algorithms for diagnosis through the recognition of soft signs [8], and predicting the risk of falls [9,10,11,12]. As for PSP, recent evidence suggests that quantitative evaluation of gait may provide further insights in both the diagnostic process and in the evaluation of disease progression [1,13,14].

Optoelectronic motion capture systems are recognized as the gold standard in the evaluation of quantitative gait parameters [15,16]. These laboratory systems are based on passive light-reflecting markers, infrared cameras to track the trajectory of markers, and force platforms to extract kinetic data [17,18]. However, the limitations of optoelectronic systems consist of high equipment costs, the need for large capture volume, technical and clinical trained personnel, and time-consuming acquisitions [19]. These aspects have recently boosted the development of wearable sensors based on Inertial Measurement Units (IMUs) for calculating gait parameters [20,21,22,23]. IMU sensors can be used in several real-life environments, both indoor and outdoor, reducing the costs of lab-based systems and the complexity of the acquisitions. Additionally, these devices are easy to wear, require less patient preparation, and, consequently, can be used by a larger number of users [24,25]. 

Recently, inertial sensors and gold standard systems have been compared in gait parameters assessment (kinematic, spatiotemporal, or both) in healthy subjects [26,27,28]. González et al. studied the reliability of the Kinovea (version 0.8.15) software, a video annotation tool designed for movement analysis, and the agreement with a three-dimensional motion system, Vicon Motion System^®^, for extracting hip, knee, and ankle joint angles parameters [29]. The same kinematic parameters were extracted by Piche et al. to compare the iSen IMU sensor with the optoelectronic gold standard system OptiTrack [30]. Finally, Bartoszec et al. compared the IMU-based MyoMotion system and the optoelectronic BTS SMART DX system, simultaneously recording the upper and lower limb joint angles [31]. Furthermore, the comparison of the above-mentioned measurement systems is also carried out to develop an algorithm for the accurate calculation of spatiotemporal gait parameters from wearable IMUs for Parkinson disease (PD) rehabilitation [32]. The Vicon Motion System^®^ was also used as gold standard reference against the system proposed in [33], based on a single RGB-D sensor to estimate the gait parameters in a limited space such as the domestic setting. Previous studies have also investigated the interoperability of IMUs sensors and optoelectronic systems in PD subjects [11,34].

In this context, the aim of the study is to evaluate the agreement between two systems for the measurement of the spatiotemporal gait parameters in patients with PSP. The BTS SMART DX optoelectronic system by BTS Bioengineering Inc. is used as reference device to the wearable Opal System by APDM Inc. To the best of our knowledge, no study has ever investigated the agreement between these two measurements systems in a population of patients with a rare disease such as the PSP. In this work, spatiotemporal parameters calculated by the two measurement systems were evaluated by means of different statistical techniques to assess their level of agreement. Since, as said above, a hard limit of the optoelectronic systems relies in the fact that they cannot be used outside the laboratorial context, the overall objective of this study is to prove that the Opal wearable system can guarantee a performance comparable to that one of the BTS SMART DX optoelectronic system.

## 2. Materials and Methods

### 2.1. Study Population and Clinical Assessment

Fifteen patients diagnosed with PSP, aged between 60 and 75 years, were recruited from the Center for Neurodegenerative diseases (CEMAND) at the University of Salerno, Italy, between January 2021 and January 2023. The diagnosis was established using the Movement Disorders Society clinical criteria [1], and all patients met the criteria for either probable or possible PSP [32]. Exclusion criteria included patients with gait requiring bilateral assistance, dementia according to the Diagnostic and Statistical Manual of Mental Disorders (DSM-V) criteria, significant comorbidities possibly impacting on gait (including other neurologic disorders, orthopedic diseases, or cardiovascular/respiratory diseases and/or brain surgery). All enrolled patients were treated with levodopa with a mean Levodopa Equivalent Daily Dose (LEDD) of 322.00 ± 177.13 [35]. The protocol was approved by the local ethics committee and all patients were enrolled upon signing a written informed consent form.

Disease severity was evaluated with the PSP-RS, administered by expert clinicians. The scale is composed of 28 items, distributed into 6 domains: history, mentation, bulbar, ocular motor, limb motor, gait/midline. The total score ranges from 0 to 100, where 100 represents the more severe outcome [4].

Demographic and clinical data are shown in Table 1.

### 2.2. Gait Analysis Systems

Two different systems were compared for gait analysis in PSP patients: the BTS SMART DX-400 System (BTS Bioengineering Corp., Milan, Italy) and the Opal System (APDM Inc., Portland, OR, USA).

**Figure 1:**
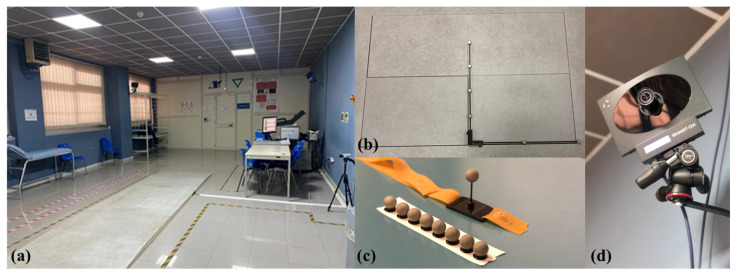
BTS Gait Lab: (**a**) Movement analysis laboratory at the University Hospital of Salerno; (**b**) Dynamometric platform; (**c**) Retro-reflective passive markers; (**d**) Infrared-camera.

The BTS SMART DX-400 is an optoelectronic system made up of six infrared digital cameras that record at a sample frequency of 100 Hz, two dynamometric platforms, twenty-two retro-reflective passive markers (each one with a diameter of 14 mm), two video cameras, and an elaborator, as shown in Figure 1. For data acquisition, the Davis protocol [36] was applied, consisting of the following steps:

- anthropometric measurements,
- positioning of passive markers on patients’ body (from the feet to the shoulders) according to the Davis protocol,
- standing phase,
- walking phases.

Then, the acquired data were transferred to the elaborator using a dedicated proprietary software, able to store and compute spatiotemporal parameters, as reported in other studies [13,37,38].

**Figure 2:**
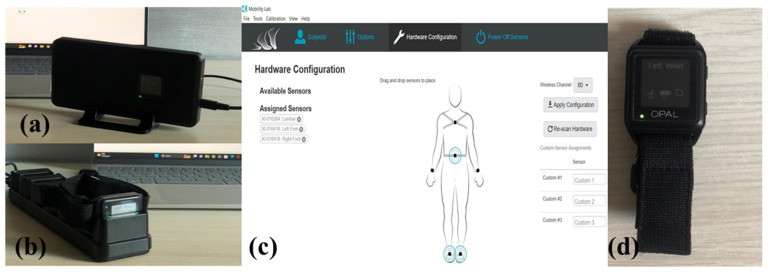
Opal System: (**a**) Access Point; (**b**) Docking Station; (**c**) Mobility Lab software; (**d**) Opal sensor.

The Opal System is a wearable inertial system made up of three IMU sensors, each one containing three-axes accelerometer with 14 bits resolution and selectable ±16 g or ±200 g ranges, three-axes gyroscope with 16 bits resolution and ±2000 deg/s, and three-axes magnetometer with 12 bits resolution and ±8 Gauss range. Opal sensors were placed on the patients’ body by means of straps, including one sensor on each foot and one sensor on the lumbar region. Thanks to the Access Point, sensors were wirelessly connected by Bluetooth to a laptop equipped with the Mobility Lab (version 4) software capable of processing movement-related data. In addition, the Docking Station was used for charging and configuring the sensors. Figure 2 illustrates the Opal Systems parts.

### 2.3. Study Protcol

Gait analysis tasks were performed in the movement analysis laboratory at the University Hospital of Salerno. First, all patients were trained to walk at their normal speed on a 10 m straight pathway four times; overall, a straight path of 40 m has been considered. Then, the four walking phases were averaged. The trials were recorded by the optoelectronic system BTS SMART DX. Before conducting the analysis with the Opal sensors, patients rested for 15 min to allow them to perform the new test without feeling tired. After such resting time, patients repeated the trials on a straight path for 10 m, walking at their normal speed for two minutes while wearing the Opal sensors. All patients were evaluated more than once.

The spatiotemporal parameters analyzed were as follows:

- Cadence (steps/minute): stepping rate.
- Cycle duration (s): duration of a complete gait cycle.
- Speed (m/s): walking speed.
- Stance phase (% gait cycle time): average percentage of a gait cycle that either foot is on the ground.
- Swing phase (% gait cycle time): average percentage of a gait cycle that either foot is off the ground.
- Stride length (m): distance between two consecutive foot falls at the moments of initial contacts.

For the current analysis, the spatiotemporal parameters from the trials were averaged for each subject in order to obtain the mean value of both sides (right and left).

### 2.4. Statistical Analyses

All statistical analyses were performed using the SPSS^®^ (version 28, IBM Corp., Armonk, NY, USA) and MATLAB (version 2021b, Mathworks Inc., Natick, MA, USA) software. In order to evaluate the agreement between the optoelectronic system (BTS SMART DX-100, BTS Bioengineering Inc., Milano, Italy) and the inertial sensors (Opal, APDM Inc., Portland, OR, USA), a two-tailed paired test was carried out. The paired data test, in its parametric and non-parametric form according to the Shapiro–Wilk normality test, was used to identify statistically significant differences between the means or the medians of the spatiotemporal variables measured by the two systems [39]. The absence of a statistically significant difference implies an overall agreement between the variables.

Passing–Bablok (PB) linear regression analysis was employed to evaluate the presence of systematic errors in the measurements. In particular, the slope of the regression line indicates a proportional systematic error, while the intercept of the regression line with the *y*-axis indicates a constant systematic error; such errors have a physical meaning whenever the slope and the intercept 95% confidence intervals contain the 1 and 0 values, respectively. The PB method was applied after verifying the linear relationship between the measurements [34,40].

The Bland–Altman (BA) method is a visual representation of the agreement between the two systems by means of a scatterplot in which the mean of the two measurements is plotted on the *x*-axis and the difference between the measurements is represented on the *y*-axis. To obtain the limits of agreement, defined as the mean of the differences ±1.96 times the standard deviation (i.e., 95% of the confidence interval), the mean of differences (bias) and the standard deviation of differences were calculated. In this context, the presence of errors can be assessed through the bias distance from the 0 value, which implies a constant systematic error, the width of the limits of agreement and any trends in the distribution of data around the bias [29,41]. In particular, possible errors could be a fan-shaped distribution within the limits of agreement, known as heteroscedasticity of variance, a non-causal but systemic distribution in absolute value, or a linear distribution of points which determine a systematic proportional error [42,43].

In all the performed analyses, the uncertainty level was set at α = 0.05.

## 3. Results

Table 2 shows the paired data test results in terms of *p*-value. Additionally, Table 2 reports the mean and standard deviation values for each spatiotemporal variable for both systems and test types, parametric or non-parametric, depending on the normality test.

The PB linear regression results of cadence, cycle duration, and speed, illustrated in Table 3, exhibited a confidence interval containing 1 for the slope and 0 for the intercept. Other parameters deviated from such values.

The results of the BA analysis for each spatiotemporal parameter included in the study are shown in Table 4 in terms of bias (the difference between two measurement systems), the 95% lower and the upper bounds of bias, and the BA lower and upper bounds, known as limits of agreement. The bias approached zero, and the bias limits containing the zero value demonstrate a good agreement between the measured parameters. 

**Figure 3:**
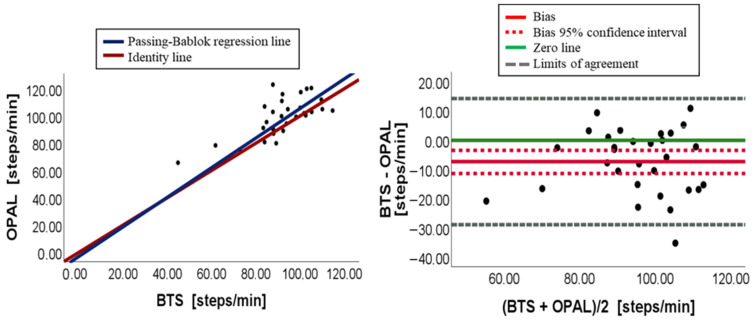
Cadence. On the left, scatter plot with PB regression line and identity line (BTS = OPAL). On the right, BA plot (average vs. difference).

**Figure 4:**
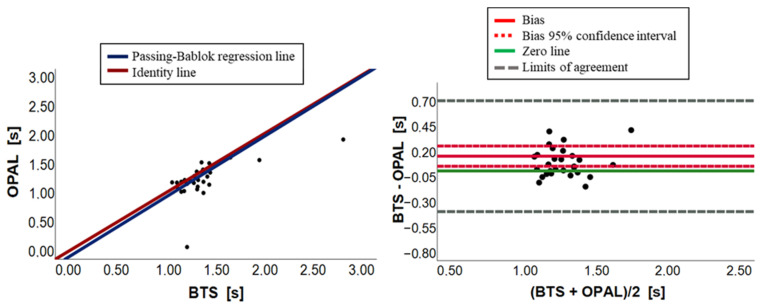
Cycle duration. On the left, scatter plot with PB regression line and identity line (BTS = OPAL). On the right, BA plot (average vs. difference).

**Figure 5:**
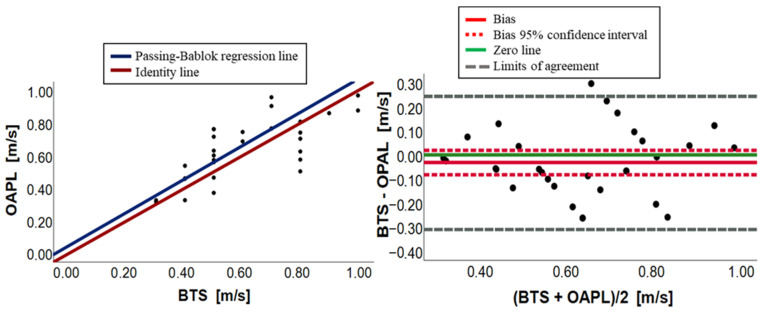
Speed. On the left, scatter plot with PB regression line and identity line (BTS = OPAL). On the right, BA plot (average vs. difference).

**Figure 6:**
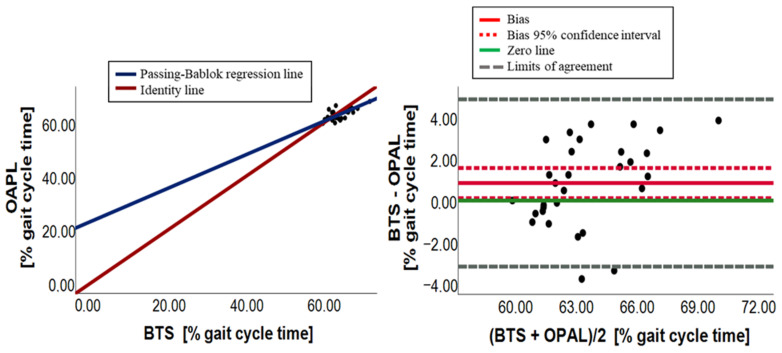
Stance phase. On the left, scatter plot with PB regression line and identity line (BTS = OPAL). On the right, BA plot (average vs. difference).

**Figure 7:**
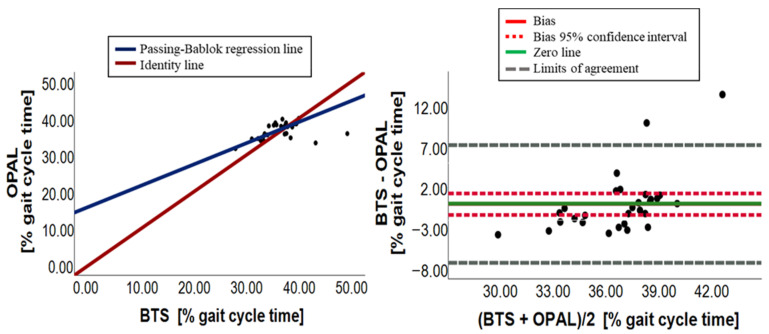
Swing phase. On the left, scatter plot with PB regression line and identity line (BTS = OPAL). On the right, BA plot (average vs. difference).

**Figure 8:**
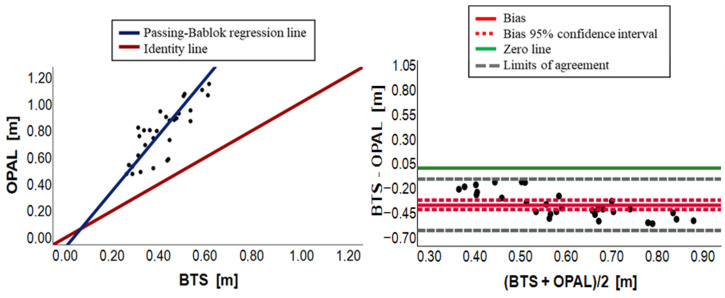
Stride length. On the left, scatter plot with PB regression line and identity line (BTS = OPAL). On the right, BA plot (average vs. difference).

Figure 3, Figure 4, Figure 5, Figure 6, Figure 7 and Figure 8 show, in pairs, the PB plot and the BA graph for all parameters (i.e., cadence, cycle duration, speed, stance phase, swing phase, and stride length).

## 4. Discussion

The current study aimed to compare the performance of Opal wearable sensors by APDM with the BTS SMART DX system in assessing spatiotemporal parameters in PSP patients during walking. Among all the spatiotemporal parameters evaluated, gait speed showed a perfect agreement between the two measurement systems, meaning it can be interchangeably evaluated with either of them. Cadence and cycle duration showed a very close agreement due to the presence of a constant systematic error, which could be removed by zeroing the bias. On the other hand, constant and proportional systematic errors were detected for the remaining parameters, meaning those cannot be interchangeably evaluated by the two systems. 

Regarding speed, first, the overall agreement was demonstrated by the Wilcoxon test with no significance between the groups (*p*-value = 0.192). The agreement was then confirmed by the PB analysis (Table 3 and Figure 5) with the slope (m = 1.02) and the intercept (q = 0.05) values of regression line close to 1 and 0, respectively, and their 95% confidence intervals containing such values. Moreover, points were equally distributed around the identity line (OPAL = BTS), as shown in Figure 5 on the left. The BA analysis in Table 4 showed the presence of a small bias equal to −0.03 and its 95% confidence interval contained the 0 value. In the BA plot, points were randomly distributed around the 0 line (Figure 5). Therefore, even though there is a very small bias, there is an absolute agreement between the two measurement systems regarding speed.

Concerning cadence, the paired data test showed a significant overall difference (*p*-value equal to 0.002), with Opal overestimating the parameter. This result was confirmed by the PB plot (Figure 3) in which the majority of points were over the identity line (OPAL = BTS). Additionally, the slope of the PB regression line was 1.09 and its 95% confidence interval (0.74–1.81) included the 1 value, assuring the absence of a proportional systematic error, while the intercept value was equal to −3.02 with a quite large 95% confidence interval (−67.89–30.17) containing 0. Nevertheless, there may be a constant systematic error due to the width of the confidence interval. Indeed, the BA analysis, illustrated in Table 4, confirmed the presence of a constant systematic error for the presence of a bias (bias = −7.43), which was quite different from 0, and its 95% confidence interval did not include 0, as shown in Figure 3. 

Similarly, as for the cadence parameter, Wilcoxon test for cycle duration showed a statistical overall difference (*p*-value equal to 0.003). In this case, the Opal system underestimated the evaluation of the cycle duration variable (Table 2 and Figure 4). The slope and the intercept of the PB regression line were close to 1 and 0, respectively (m = 1.03; q = −0.10), and their 95% confidence intervals contained the 1 and 0 values, which allows to suppose the presence of agreement. Despite this, the presence of a constant systematic error has been detected by the BA analysis (Table 4 and Figure 4). Indeed, there was a small bias, equal to 0.15 with a confidence interval, ranging from 0.05 to 0.25. The BA plots for cadence and cycle duration illustrated a random distribution of points around the zero-line, confirming the presence of only a constant systematic error. In conclusion, due to a constant systematic error, there is no agreement between the BTS systems and wearable sensors for both cadence and cycle duration parameters, which could be achieved by zeroing the bias. 

Concerning the stance phase, the paired data test exhibited a significant overall difference (*p*-value = 0.037), implying the absence of an overall agreement. The slope of the PB regression line was 0.63 and its 95% confidence interval ranging from 0.46 to 0.85 did not include the 1 value, showing the presence of a proportional systematic error; the intercept was 23.09 with a 95% confidence interval that did not contain the 0, indicating the presence of a constant systematic error; the presence of such errors has emerged also from the analysis of BA (Table 4 and Figure 6). The bias of the stance phase was close to 0 (bias = 0.84) but its 95% confidence interval did not include the 0 value. Furthermore, the points distribution followed a linear trend around the bias, as shown in Figure 6. In conclusion, there is no agreement between the two measurement systems due to the presence of a constant systematic error and a proportional systematic error.

Regarding the swing phase, no significance between groups was found (*p*-value = 0.159) from Wilcoxon test, suggesting the presence of an overall agreement. Nevertheless, the slope and the intercept of the PB regression line (m = 0.58; q = 15.96) were far from the identity line values and their 95% confidence intervals did not include 1 and 0, respectively, showing the presence of a proportional and a constant systematic error (Table 3). The BA analysis, illustrated in Table 4, confirmed the presence of both errors for the presence of bias (bias = −0.05), whose 95% confidence interval contained 0, and for the linear distribution of points around the bias (Figure 7). Therefore, due to a constant systematic error, we can conclude that there is no agreement between BTS and Opal systems for the swing phase.

Regarding stride length, the T-Student paired test showed a statistical overall difference (*p*-value < 0.001) with Opal overestimating the parameter (Table 2). This result was confirmed by the PB scatter plot in Figure 8. The PB analysis, in Table 3, illustrated the presence of a proportional systematic error with a slope value far from 1 and its 95% confidence interval, ranging from 1.64 to 2.69, which did not include it. The intercept of the PB regression line was −0.05 and its 95% confidence interval (−0.28–0.13) contained the 0 value, assuring the absence of a constant systematic error. The absence of a constant systematic error was not confirmed by the BA analysis due to the presence of a small bias equal to −0.37, and its 95% confidence interval did not include the 0, as shown in Table 4. In addition, the BA plot in Figure 8, where the linear trend of points around the bias is shown, implies the presence of a proportional systematic error. In conclusion, there is no agreement between the two measurement systems due to the presence of a constant systematic error and a proportional systematic error. Results are summarized in Table 5.

Recently, Zago et al. compared the BTS SMART DX optoelectronic system to the G-Sensor wearable system, assessing spatiotemporal parameters in patients’ diagnosis with PD [11]. The variables cadence, stride length, stride duration, step duration, stance duration, swing duration, and double support showed no statistical significance from Wilcoxon rank-sum test (*p*-value > 0.05) except for velocity showing a *p*-value equal to 0.01. The Spearman correlation analysis obtained a good correlation between the two measurement systems, reaching high correlation coefficient values (from 0.64 to 0.91) except for step duration that achieved a correlation coefficient value equal to 0.41. High values, over 10% of the mean absolute error, were obtained for stride length, step duration, stance, and double support duration. The overall results reached by the IMUs system were sufficiently accurate for spatiotemporal parameters evaluations, showing a discrepancy with our results. The same optoelectronic system in comparison with an IMU-based system was used in another study for kinematic gait parameters assessment in healthy subjects [31]. A high relationship between the two measurement systems was obtained by the correlation coefficient r for shoulder flexion-extension, ankle dorsi-plantar flexion, elbow flexion-extension, knee flexion-extension, hip abduction-adduction, and hip flexion-extension. The BA analysis results showed that the IMU-based system overestimates or underestimates the joint angles measurements, reaching bias values greater than 10 degrees. Moreover, Vítecková et al. achieved results comparable to ours by means of different measurement systems (the GAITRite optoelectronic system and the wearable G-Walk system) [34]. Compared to our study, other parameters were additionally evaluated, namely stride duration, double, and single support. Cadence, speed, stride length, and stride duration demonstrated no statistical differences from the paired *t*-test. The PB analysis showed slope and intercept values very close to 1 and 0 for cadence and stride duration, whereas the interclass correlation coefficient exhibited high correlation value (r > 0.75) for speed, stride duration, cadence, and stride length. In particular, the agreement was reached for the speed variable in both studies, whereas parameters that could not be considered interchangeable for the two systems and for both studies were stance and swing phase.

The use of gait analysis systems in movement disorders is of utmost importance for regular outpatient follow-ups and for research purposes. Recent evidence demonstrated that Opal wearable sensors present a good correlation with PSP-RS and can effectively detect disease severity and progression in PSP [14,44,45]. Wearable sensors present several advantages compared to more sophisticated systems gathering spatiotemporal parameters of gait for both clinical and research settings. First, set-up and placement are swift and straightforward and only require a short training. Then, the report is promptly provided by the parent company software and normal and previous evaluation values are always shown on the output, making it easy to be interpreted even by non-expert clinicians. As a matter of fact, in just a few minutes, the clinician can have a complete overview of patients gait impairment. Moreover, since PSP patients usually suffer from a severe and rapidly progressive disability, it could be easier for them to use wearable sensors at home or in their outpatient clinic rather than reaching a specific movement analysis laboratory. From a research point of view, wearable sensors can be easily added to a protocol evaluating the efficacy of disease treatments and implemented even in research centers lacking a specific gait lab with more sophisticated instruments.

One limitation of the present study concerns the acquisitions that could not be conducted at the same time due to the incompatibility of the two systems (positions of sensors and markers in the same points of the body and possibility of infrared ray reflection), but they were conducted in the same conditions to reduce the risk of bias at the minimum. Other studies in the literature emphasized the small sample size [11,28,31,32,34] in addition to the fact that patients walk straight paths in lab-based or clinical settings; nevertheless, our study focused on a rare disease, which means that our sample size is consistent with other studies investigating PSP [11,13]. It could also be useful for evaluating a cohort of patients with several gait-related conditions. Furthermore, the different algorithms implemented in the measurement systems can introduce discrepancies in the measurement of parameters.

## 5. Conclusions

This study proposed a comparison between the wearable Opal system, and the optoelectronic lab-based system, BTS SMART DX, for assessing gait-related parameters in PSP patients. The agreement study conducted between the two measurement systems does not allow the conclusion that the two devices can be used with a complete interchangeability, due to the presence of two types of errors: a constant systematic error detectable with bias zeroing (cadence and cycle duration), and a proportional error (stance phase, swing phase and stride length). This result does not apply to the speed variable that showed perfect agreement between the two measurement systems. This is a first step in defining the reliability of a wearable sensor system compared to the actual gait analysis gold standard. Given the increasing importance of implementing wearable technologies in both clinical and research settings, the feasibility for non-expert clinicians and the known sensitivity of the Opal system, the present data represent a further step forward the implementation of quantitative evaluation of gait in PSP patients. A further development could be focused on the need to study the algorithm to detect gait events underlying systems by reducing the offset.

## Figures and Tables

**Table 1:** Patients’ anthropometrics and clinical data (n = 15; 5 females and 10 males).

Clinical Parameters	Mean ± SD
Age (years)	68.13 ± 4.97
Disease duration (years)	3.13 ± 2.07
PSP-RS limb	3.33 ± 2.29
PSP-RS gait/midline	5.20 ± 4.78
LEDD (mg)	322.00 ± 177.13
BMI (kg/m^2^)	27.48 ± 3.84

Abbreviations: BMI = Body Mass Index; LEDD = Levodopa Equivalent Daily Dose; PSP-RS = Progressive Supranuclear Palsy Rating Scale.

**Table 2:** Two-tailed paired test analysis to test the overall agreement between each couple of parameters of the systems.

Spatiotemporal Parameters	BTS SMART DX System	Opal System	*p*-Value	Test Type
Cadence (steps/min)	91.72 ± 14.23	99.14 ± 14.28	0.002	Wilcoxon test
Cycle duration (s)	1.36 ± 0.33	1.21 ± 0.30	0.003	Wilcoxon test
Speed (m/s)	0.61 ± 0.20	0.65 ± 0.19	0.192	Wilcoxon test
Stance phase (% gait cycle time)	63.96 ± 2.92	62.12 ± 2.11	0.037	Wilcoxon test
Swing phase (% gait cycle time)	36.83 ± 3.92	36.88 ± 2.11	0.159	Wilcoxon test
Stride length (m)	0.41 ± 0.10	0.78 ± 0.20	<0.001	*T*-Student test

**Table 3:** PB linear regression analysis.

Spatiotemporal Parameters	m *	LB_m	UB_m	q **	LB_q	UB_q
Cadence (steps/min)	1.09	0.74	1.81	−3.02	−67.89	30.17
Cycle duration (s)	1.03	0.63	1.74	−0.10	−1.04	0.40
Speed (m/s)	1.02	0.71	1.45	0.05	−0.17	0.22
Stance phase (% gait cycle time)	0.63	0.46	0.85	23.09	8.78	33.34
Swing phase (% gait cycle time)	0.58	0.32	0.74	15.96	10.13	25.68
Stride length (m)	2.10	1.64	2.69	−0.06	−0.28	0.13

* m is the slope of Passing-Bablok regression line; ** q is the intercept of Passing-Bablok regression line. Abbreviations: LB = Lower Bound of confidence interval; UB = Upper Bound of confidence interval.

**Table 4:** BA analysis applied per each couple of parameters of the systems.

Spatiotemporal Parameters	Bias	LB_b	UB_b	LB_LA	UB_LA
Cadence (steps/min)	−7.43	−11.36	−3.50	−28.95	14.10
Cycle duration (s)	0.15	0.05	0.25	−0.40	0.70
Speed (m/s)	−0.03	−0.08	0.02	−0.31	0.25
Stance phase (% gait cycle time)	0.84	0.11	1.57	−3.17	4.84
Swing phase (% gait cycle time)	−0.05	−1.37	1.27	−7.30	7.20
Stride length (m)	−0.37	−0.42	−0.32	−0.63	−0.11

Abbreviations: LA = Limit of Agreement; LB = Lower Bound; UB = Upper Bound.

**Table 5:** Agreement results.

Spatiotemporal Parameters	Level of Agreement	Error Types
Cadence (steps/min)	Very close agreement	Constant systematic error
Cycle duration (s)	Very close agreement	Constant systematic error
Speed (m/s)	Agreement	/
Stance phase (% gait cycle time)	No agreement	Constant and proportional systematic errors
Swing phase (% gait cycle time)	No agreement	Constant and proportional systematic errors
Stride length (m)	No agreement	Constant and proportional systematic errors

## Data Availability

The data presented in this study are available on request from the corresponding author. The data are not publicly available due to the privacy policy.
